# Allergic conjunctivitis

**Published:** 2017

**Authors:** Varsha M Rathi, Somasheila I Murthy

**Affiliations:** Faculty, Tej Kohli Cornea Institute, Gullapalli Pratibha Rao International Center for Advancement of Rural Eye Care, L V Prasad Eye Institute, Hyderabad, India; Faculty, Tej Kohli Cornea Institute, L.V. Prasad Eye Institute, Kallam Anji Reddy Campus, Hyderabad, India

**Figure:**
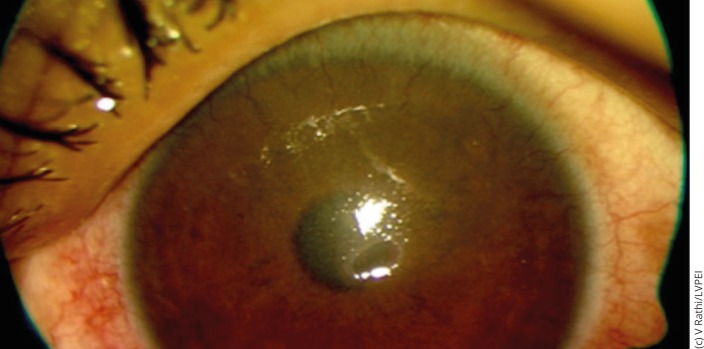
Roughness of epithelium as seen in shield ulcer

**Figure:**
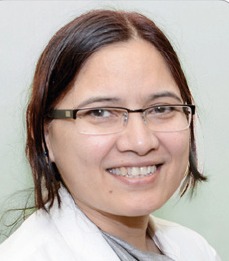
Varsha M Rathi

**Figure:**
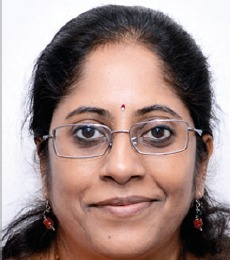
Somasheila I Murthy

The diagnosis of allergic diseases has increased in the last few decades and allergic conjunctivitis has emerged as a significant problem, which can cause severe ocular surface disease. Patients complain of itching, watering and redness. It can result in decreased quality of life, as patients with severe symptoms, if left untreated or treated poorly, may become school dropouts, unable to work outdoors and sometimes fail to sleep. The symptoms are aggravated by exposure to dry and windy climates.^1^,^2^ This article aims to provide a brief overview of the management of allergic conjunctivitis. The most important symptom of allergic conjunctivitis is itching. Table 1 lists spectrum of disorders of allergic conjunctivitis.^3^

## Epidemiology

The diagnosis of allergic conjunctivitis is on the increase. SAC and PAC accounts for 15–20% of cases of allergic conjunctivitis.^4^ The disease is more common in hot, humid tropical climates.^5^ VKC has been reported from many Asian countries e.g. Nepal, Pakistan and India.^2^,^6^,^7^ VKC and AKC may cause corneal and ocular surface involvement leading to severe visual loss. Numerous factors such as changing climates, increasing pollution, genetics, cigarette pollutants and occurrence of allergy in early childhood have been proposed as causative agents or risk factors. Signfiicant correlations have been observed with mixed pollen, thresher dust and raw cotton with allergic rhinitis and allergic conjunctivitis.^8^ Seasonal peak is seen during April to August in patients having VKC.^9^

## Classification

### Seasonal allergic conjunctivitis

This condition is common, is seen among all ages and occurs seasonally when pollen is released in May and June. Itching followed by watering and a burning sensation is seen in these patients. Sometimes, it may be associated with a running nose (allergic rhinitis or rhinoconjunctivitis). Patients may complain of sinus pressure behind the eye.

**Table 1.** Disorders of allergic conjunctivitis

Mild allergic conjunctivitis	Severe allergic conjunctivitis	Chronic microtrauma related disorders
Seasonal conjunctivitis (SAC)	Vernal keratoconjunctivitis (VKC)	Contact lens induced papillaryconjunctivitis (CLPC)
Perennial conjunctivitis (PAC)	Atopic keratoconjunctivitis (AKC)	Giant papillary conjunctivitis (GPC)

### Perennial allergic conjunctivitis

PAC has similar signs and symptoms to SAC and as the name suggests it occurs throughout the year. PAC is due to allergy to animal dander, mites and feathers. The frequency of occurrence increases as the age increases.^10^ The patients have itching, redness and swelling of conjunctiva. Corneal involvement in SAC and PAC is rare.^4^

### Vernal keratoconjunctivitis

VKC is a disease of warm climates and occurs predominantly in young males (8–12 years of age).^2^,^11^ Although VKC is more common in children, adults may also have VKC.^12^,^13^ It is a bilateral disease and may worsen with exposure to wind, dust and sunlight. These patients may have positive history of asthma or eczema. Patients present with severe itching (rubbing of eyes usually with a knuckle), redness, discharge, and photophobia. The mucus discharge is thread-like. School-going children may drop out from going to school because of severe itching and photophobia.

**Figure 1.**
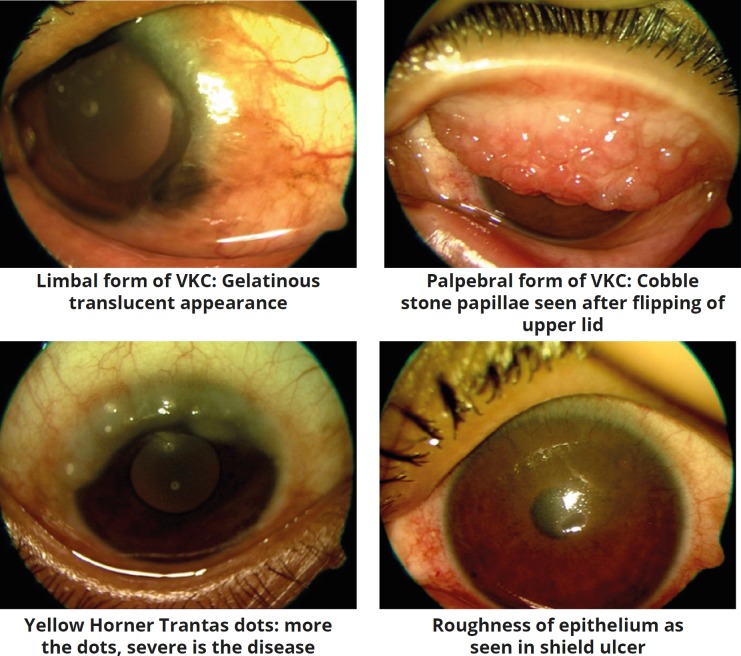
Clinical forms of VKC: Limbal or bulbar, palpebral and mixed

Three clinical forms of VKC are described: limbal or bulbar, palpebral and mixed (Figure 1). Limbal form is more common in dark skinned individuals. In Asia, the mixed form is more common compared to the limbal form, which is seen in Africans.^7^ However, studies from India and Nepal have reported that the bulbar form of the disease is common in some areas.^2^,^9^

Limbal or bulbar form may present as gelatinous thickening of the limbus, presence of papillae at the limbus and yellow Horner-Tranta's dots (Figure 1) usually at the superior limbus. These dots are seen when the disease is active and indicate severity of the disease.

The hallmark of the palpebral VKC is presence of giant papillae, which are seen on everting the upper lid – the giant papillae have a cobble stone appearance (Figure 1). This thickening of the upper lid may be associated with drooping of the lid (ptosis). Conjunctival pigmentation is common in patients having VKC.^14^

The mixed form of VKC has features of both palpebral and limbal VKC. Corneal involvement in VKC may occur as corneal epithelial punctuate keratitis, and where the epithelial erosions may coalesce and form a vernal or a shield ulcer. Presence of shield ulcer will worsen patients' symptoms and affect vision. These ulcers are oval and are usually present in the upper part of the cornea. The shield ulcers are classified based on the presence of white material at the base of the ulcer. Based on the grades of shield ulcer, the treatment options differ.^15^

### Atopic keratoconjunctivitis

AKC is a bilateral disease of ocular surface and lids, which occurs throughout life. The patients will have eczematous skin lesions of the body. The conjunctiva may have papillae or Trantas dots. Cataract formation can occur in these patients. Table 2 shows the differentiating features of VKC and AKC.

**Table 2.** Differentiating features of vernal and atopic keratoconjunctivitis

	VKC	AKC
**Age**	Young	Old
**Sex**	Males > Females	Equal ratio
**Season**	Spring	Perennial
**Duration**	Limited	Chronic
**Skin involvement**	No	Yes, extra lid fold, maceration of canthi
**Punctal stenosis**	No	Yes
**Conjunctiva**	Upper tarsal conjunctiva	Lower tarsal conjunctiva
**Conjunctival scarring**	Rare	Common
**Cornea**	Shield ulcer	Epithelial defects
**Scarring**	Peripheral	Central
**Vascularisation**	Rare	Common
**VKC: vernal keratoconjunctivitis AKC: Allergic keratoconjunctivitis**		

### Giant papillary conjunctivitis

The presence of a contact lens, ocular prosthesis or sutures may sensitise and cause trauma to the upper tarsal conjunctiva with the formation of giant papillae. Removal of these external agents will reduce the papillae. Toxic allergic reactions may also be due to drugs such as neomycin, atropine, epinephrine or preservatives in medicines such as thiomersol.^16^

### Contact hypersensitivity reactions

The pattern of involvement depends upon severity of the reaction and the site of contacts. Patients may have lid swelling, redness, chemosis, follicular reaction and later sometimes cicatrisation. The corneal involvement may be in the form of superficial punctate keratitis, pseudodendrites or grayish stromal infiltrates.^17^

## Complications

Most often, the complications are because of poor compliance to treatment on the part of patient, or inadequate control of the disease when it presents in its severe form. Common complications include dry eye, infection and corneal scar. Chronicity of the untreated disease may lead to vision threatening problems like limbal stem cell deficiency (LSCD) and secondary keratoconus due to rubbing of the eyes.

As the treatment involves use of corticosteroids, steroid-induced raised intraocular pressure and cataract have been reported in these patients.^7^ Complications may lead to irreversible visual loss in some patients.^7^ Both the complications, keratoconus and LSCD need timely surgical treatment to prevent visual malfunction.

## Diagnosis

**Figure 2.**
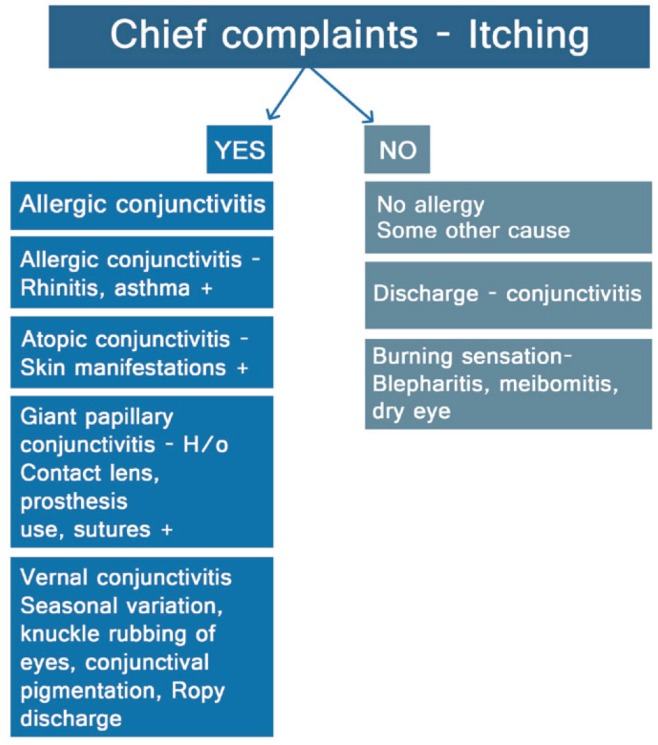
A guide to aid diagnosis of allergic conjunctivitis

Appropriate management of allergic conjunctivitis needs a correct diagnosis. Figure 2 gives a guide for such diagnosis and ways to differentiate from other causes of red eyes. Presence of itching is a hallmark of ocular allergy.

## Management

Though some authors have described management protocols, there are no universally accepted protocols of management for allergic eye diseases.^11^,^12^ Various drugs are available and the treatment options vary based on the severity of the disease. It is important to avoid any known allergen or reduce exposure to it by using wrap around glasses, by changing the environment, replacing allergen harbouring items such as pillows and carpets. However, such recommendations may be challenging for patients. In addition, cool compresses can be done to prevent rubbing of the eye. Ocular lubricating eye drops can be used to dilute the inflammatory agents in tears and wash away the allergen to reduce itching and to prevent further worsening of symptoms.^19^

The mainstay of treatment is the use of lubricants, anti-histamines and mast cell stabilisers.^16^,^20^ These are indicated in all forms of disease. Steroids are to be given under proper medical care when the cornea is involved or the disease is very severe with itching.

Overuse of corticosteroids may cause steroid induced cataracts and glaucoma and may result in blindness. The drugs that are used are:

**Mast cell stabilisers:** disodium cromoglyacate (not effective in acute stages), Nedocromil and Lodoxamide

**Antihistamines:** ketotifen, dual acting drugs such as olapatadine, azelastine, epinastine and bepostatine. Immediate symptomatic relief is possible with azelastine and epinastine, which are currently preferred.

**Corticosteroids:** such as prednisolone are given for a short duration during acute allergic disease; oral steroids or supratarsal injection of corticosteroids is required if the disease is severe.

**Nonsteroidal anti-inflammatory agents (NSAIDS):** ketorolac, diclofenac can be added to antihistamines. Steroid sparing agents such as Cyclosporine A, Tacrolimus are effective in severe AKC and VKC.

## Conclusion

From a public health perspective, the number of patients being diagnosed with allergic conjunctivitis is increasing. However, not many studies are available from South East Asia, which give a complete picture of allergic eye disease. Severe conjunctivitis such as VKC, being a disease of the young may increase the number of school dropouts in these countries. Economic costs for patients are high, sometimes necessitating the need for medications to continue for years.^21^,^22^ Management of the disease is very challenging and a multipronged approach with well-trained primary and secondary care personnel to educate patients or parents about the disease, especially about good general hygiene; avoidance of allergens; cold compression; change of environment; and judicious use of corticosteroids may improve ocular health in patients by leaps and bounds.
